# Perspectives and pregnancy outcomes of maternal Ramadan fasting in the second trimester of pregnancy

**DOI:** 10.1186/s12884-019-2275-x

**Published:** 2019-04-15

**Authors:** Kolsoom Safari, Tiran Jamil Piro, Hamdia Mirkhan Ahmad

**Affiliations:** 1 Department of Midwifery, College of Nursing, Hawler Medical University, Erbil, Iraq; 2 Department of Basic Science, College of Basic Science, Hawler Medical University, Erbil, Iraq

**Keywords:** Ramadan, Fasting, Pregnancy, Gestational diabetes

## Abstract

**Background:**

There are controversies over the effects of Ramadan fasting on pregnancy outcomes, and women’s perspectives of fasting are diverse. This study aimed to assess the perspectives and pregnancy outcomes of maternal Ramadan fasting in the second trimester of pregnancy.

**Methods:**

A case-control study was conducted at Hawler Maternity Teaching Hospital of Erbil, Iraq from October 2017 to January 2018. Out of 301 participating women, 155 fasted during the second trimester of their current pregnancy, while the remaining 146 did not. Mothers were asked concerning their fasting behaviors and perception of fasting during pregnancy. The main outcomes of this study were gestational diabetes, preterm labour, preeclampsia, low birth weight, Apgar score, height, weight, and head circumference of the newborn.

**Results:**

About 80% of the women in the fasting group fasted for 21–29 days during Ramadan, out of whom 38.7% completed fasting for the entire Ramadan period. The results revealed that the decision to fast during pregnancy was negatively associated with the mother’s educational level and occupation. Weight gain during pregnancy in the fasting women was approximately 0.4 kg less than those who did not fast. The incidence of gestational diabetes was 2.6% in the fasting women, while it was 8.3% in the non-fasting mothers (*P* = 0.02). Regression analysis showed that women who did not fast during the second trimester of pregnancy were 1.51 times more likely to develop gestational diabetes [odd ratio (OR) 1.51; 95% confidence intervals (CI) 0.06, 0.74, *P* = 0.01]. It was also found that among the women in the fasting categories, those who fasted for 21–29 days during pregnancy had a lower risk of gestational diabetes compared to the other groups. More than half of the mothers in the fasting group (60%) perceived that fasting during pregnancy was compulsory for healthy and non-healthy women, comparing with those who did not fast.

**Conclusion:**

It was found that fasting during the second trimester of the pregnancy decreased the risk of gestational diabetes and excessive weight gain during pregnancy. Most of Iraqi women did not fully recognize their right to be exempted from fasting during pregnancy by the Islamic law.

**Electronic supplementary material:**

The online version of this article (10.1186/s12884-019-2275-x) contains supplementary material, which is available to authorized users.

## Background

According to the Islamic laws, all adults and healthy Muslims are obliged on fast during the holy month of Ramadan [1]. The routine of Ramadan fasting obligates abstinence from all food and liquid items from the crack of dawn until sunset. Seasonal and geographical conditions can influence daily fasting duration which may vary from 11 to 18 h per day [2].

From a religious perspective, healthy pregnant women are exempted from fasting if they believe that their health or their fetus’s health might be negatively affected; however, this blurred guidance could make some women believe that fasting is an obligation, and that they need to practice fasting during Ramadan [3]. Knowledge should be gathered on the mothers’ perception of fasting since a high proportion of pregnant Muslim women adhere to fast each year, and there is still inconsistent information regarding the influences of the fasting on pregnancy outcomes. Majorities of the Iraqi population (97%) are Muslims [4]; therefore, health care providers need to be provided with useful information and adequately counsel pregnant Muslim women regarding Ramadan fasting [5].

Over the past decade, Ramadan has fallen in summer months when the temperature reaches 50 °C at midday, and the number of fasting hours in Iraq increases [6]. During the fasting hours and after breaking the fast, metabolic condition of the body could be influenced as a consequence of change in the pattern and amount of activity, meals and fluid intake, and even sleeping hours during this month [7, 8]. This situation may be different for pregnant women and could affect health conditions of mothers and fetuses. According to the results of both human and animal studies, prolonged periods without food intake during pregnancy were found to increase the risk of elevated maternal corticotropin-releasing hormone concentrations with possible maternal and fetal health consequences [3]. Also, in utero exposure to Ramadan has long-term implications for the health of offspring, possibly through fetal programming [9]. Research has also revealed that Ramadan fasting during pregnancy increases the risk of lower birth weight, hyperemesis gravidarum, and decreased fetal breathing movements [10–13]. On the other hand, many other studies found that fasting has no effect on intrauterine growth, birth weight, birth-time indices, gestational diabetes, preterm birth, and preeclampsia. Predominantly, results of the studies examining the effects of fasting on mothers and newborns are not homogenous; therefore, further research should be conducted to attain valid findings [3].

Although Muslim women who adhere to fast in Ramadan every year encompass a considerable part of Iraq population, there is scarce evidence on their fasting behaviors during pregnancy. Additionally, to provide these women with appropriate care during pregnancy, it is substantial to know their perspective regarding Ramadan fasting. The aim of the present study was to assess the perspectives and pregnancy outcomes of maternal Ramadan fasting in the second trimester of pregnancy.

## Methods

A case-control study was conducted at Hawler Maternity Teaching Hospital of Erbil, Iraq from October 2017 to January 2018. Erbil lies 80 km (50 miles) east of Mosul, and is the capital of the Kurdistan Region of Iraq. The predominant religion in the Governorate of Erbil is the Sunni branch of Islam, and the main ethnic group is Kurd [14].

Sample size of this study has been calculated based on the similar study which was conducted in Bradford Royal Infirmary UK [15]. Considering the proportion of women with gestational diabetes and preterm labour in fasting and non-fasting groups in the UK study, it has been calculated to include 142 women in each group in our study at α = 0.05 and b = 0.20. Finally, 301 healthy pregnant Muslim women within the age range of 18–35 years with live singleton birth were participated; 155 women fasted during the second trimester of their current pregnancy and 146 did not. Women in both groups were homogenized in terms of their age and number of para. Eligible women who attended the delivery room of Hawler Maternity Teaching Hospital for birth were invited to join the study and if agreed, they signed written informed consent to participate in this study. Approval (Ref 32 N/2017) was provided by College of Nursing Research Ethics Committee at Hawler Medical University.

In 2017, Ramadan spanned from 27th of May through 25th of June, a total period of 29 days with fasting times of up to 18 h per day. The first question asked whether women fasted during the second trimester of their current pregnancy or not. Then women in the fasting group were asked for the number of days they had fasted, which was categorized as 1–10 days, 11–20 days, and 21–29 days.

A questionnaire which was completed by researcher was designed based on similar studies conducted on the same subject [3], translated by a linguistic expert in Kurdish language for ease of understanding and administration to the study population, and finally validated by relevant experts (see Additional file 1). In addition to the baseline questionnaire regarding the mothers’ age, education level, income, number of para, and BMI before pregnancy, questions concerning their fasting behaviors and perceptions of fasting during pregnancy were asked from the participants. The women were given several alternative answers regarding their perceptions of Ramadan fasting; however, if their answer were not included in the questionnaire, the women were allowed to provide their own answer.

### Outcomes

The outcomes assessed in this study were gestational diabetes, preterm delivery, preeclampsia, low birth weight, fetal weight, height and head circumference at birth, fetal Apgar score, and birth weight as they had previously been found to be related with Ramadan fasting [3, 10, 16–18]. Preterm labor was defined as the presence of uterine contractions of sufficient frequency and intensity to effect progressive effacement and dilation of the cervix prior to term gestation, occurring at 20–37 weeks’ gestation [19].

Gestational diabetes mellitus (GDM) was defined as any degree of glucose intolerance with onset or first recognition during pregnancy. In this study, Glucose Challenge Test with 50 g glucose was performed for screening of GDM, and result of > 7.8 mmol/l followed by 3-h Oral Glucose Tolerance Test for confirmation of diagnosis [20]. Preeclampsia was defined as a disorder of widespread vascular endothelial malfunction and vasospasm that occurs after 20 weeks’ gestation and can present as late as 4–6 weeks postpartum. It was clinically defined by hypertension and proteinuria, with or without pathologic edema [21].

### Statistical analysis

The collected data were analyzed using SPSS IBM v.21 [22]. Descriptive relationships between demographic variables and fasting behavior were assessed using means and SD for continuous variables, whilst categorical variables were described using proportions. The relationship between fasting behaviors and pregnancy outcomes, including gestational diabetes mellitus (GDM), preterm birth, preeclampsia, anthropometrics of newborn, low birth weight, and 5th minute Apgar score were examined using t-test and Chi-square. To analyze the relationship between fasting categories and pregnancy outcomes, Chi-square and one-way ANOVA (One-way analysis of variance) test were used. In the regression model, the effect of fasting behavior on pregnancy outcome was examined by adjusting age, maternal education, maternal occupation, number of para, weight gain during pregnancy, and the BMI before pregnancy. To determine the differences between the fasting categories Fisher’s Least Significant Difference, post-hoc test was used. This study had 60% power at a 95% level of confidence to detect 24% difference in GDM, and 72% power to detect 20% differences in maternal weight gain during pregnancy between fasting and non-fasting women.

## Results

Approximately 55% (*n* = 155) of the participants reported fasting during Ramadan in 2017. Of these women who fasted, 38.7% fasted for the whole period of Ramadan. The mothers’ relatives comprised the main source for them to receive information regarding fasting during Ramadan. The proportion of women who fasted for 21–29 days, 11–20 days, and 1–10 days during pregnancy were 132 (85.16%), 14 (9.03%), and 8 (5.16%), respectively.

Table 1 describes the differences in maternal characteristics by fasting behavior. Women in the fasting group did not differ from the non-fasting group with respect to all characteristics, except for their educational level and employment condition. In both groups, the proportions of primiparous women were approximately similar. The results showed that women who had educational qualifications of equal or greater than secondary school were less likely to fast during pregnancy (*P* = 0.005). Additionally, a higher number of the non-fasting women (20.8%) worked compared with the fasting women (11%).

**Table 1:** Maternal characteristics stratified by maternal fasting category

	Fasted (*n* = 155)	Non (*n* = 144)	*P* value
Mothers’ age (year)	27.87 ± 6.51	27.08 ± 6.37	P = 0.7^a^
Mothers’ education level			
• Primary school	102 (65.8%)	67 (46.5%)	P = 0.005^b^
• Secondary school	36 (23.2%)	50 (34.7%)	
• Academic education	16 (10.3%)	27 (18.8%)	
Residency			
• Erbil	103 (66.5%)	100 (69.4%)	P = 0.25^b^
• Suburban	52 (33.5%)	42 (29.2%)	
Maternal employment			
• Not working	138 (89%)	112 (77.8%)	P = 0.01^b^
• Working	17 (11%)	30 (20.8%)	
Number of gravid			P = 0.42^b^
• 1–2	133 (85.8%)	128 (88.9%)	
• 3–4	22 (14.2%)	16 (11.1)	
Number of para			P = 0.27^b^
• 0	2 (1.3%)	0	
• 1	108 (69.7%)	108 (75%)	
• 2	45 (29%)	35 (25%)	
Number of miscarriage			P = 0.53^b^
• 0	110 (71%)	107 (74.3%)	
• 1–2	40 (25.8%)	35 (24.3%)	
• 3	5 (3.2%)	2 (1.4%)	
Monthly income* (USD)	383.58 ± 203.39	360.44 ± 184.36	P = 0.43^a^
Mothers’ BMI (before pregnancy)			
• Underweight	4 (2.6%)	9 (6.3%)	
• Normal	84 (55.3%)	83 (58%)	P = 0.32^b^
• Overweight	50 (32.9%)	42 (29.4%)	
• Obese	14 (9.2%)	9 (6.3%)	
• Maternal weight gain during pregnancy (kg)	10.84 ± 4.69	11.29 ± 3.06	0.009^a^
- Where does she collect information regarding Ramadan fasting in pregnancy?			
• Mullah	49 (31%)	45 (31.2%)	P = 0.29^b^
• Relatives	75 (48.4%)	67 (46.5%)	
• Obstetrician	13 (8.4)	15 (10.4%)	
• Nurse	0	4 (2.8%)	
• Media	18 (11.6%)	13 (9%)	
- Number of days mothers fasted			
• Non	–	144 (100%)	
• 1–10 days	8 (5.16%)	–	
• 11–20 days	14 (9.03%)	–	
• 21–29 days	132 (85.16%)	–	

- Data are presented as mean (standard deviation) in case of continuous variables and n (%) in case of frequencies

* Monthly income is the total family income per month, which is calculated by converting it from Iraqi Dinar (IQD) to United States Dollar (USD)

^a^ Independent samples t-test

^b^ Chi-square test

The fasting and non-fasting mothers were not statistically different in terms of their BMI before pregnancy. Weight gain during pregnancy was 11.29 ± 3.06 kg (mean ± SD) in the non-fasting mothers and 10.84 ± 4.69 kg (mean ± SD) in the mothers who fasted during pregnancy (*P* = 0.009).

The results were found to be statistically significant for gestational diabetes when cross-tabulation and t-test were used to find the association between fasting and pregnancy outcomes. Prevalence of gestational diabetes was 2.6% in the mothers who fasted during pregnancy and 8.3% in the non-fasting mothers (*P* = 0.02). No association was found between other maternal and neonatal outcomes like preterm labour, preeclampsia, mode of delivery, and anthropometrics measurement of the newborns with fasting (Table 2).

**Table 2:** Relationship of maternal and neonatal outcome of pregnancy with fasting

	Fasted (*n* = 155)	Non (*n* = 144)	*P* value
- Mode of delivery			
• Vaginal delivery	66 (42.6%)	74 (51.4%)	
• Cesarean section	86 (55.5%)	42 (29.2%)	0.37^a^
- Gestational diabetes			
• Yes	4 (2.6%)	12 (8.3%)	0.02^a^
• No	151 (97.4%)	132 (91.7%)	
- Preeclampsia			
• Yes	23 (14.8%)	18 (12.5%)	0.55^a^
• No	132 (85.2%)	126 (87.5%)	
- Preterm labour			
• Yes	7 (4.5%)	5 (3.5%)	0.64^a^
• No	148 (95.5%)	139 (96.5%)	
- Low Birth Weight	6 (3.87%)	4 (2.77%)	0.59^b^
- Fetal birth weight (kg)	3.47 (1.68)	3.35 (0.52)	0.17^b^
- Fetal height (cm)	47.11 (5.1)	47.23 (2.79)	0.07^b^
- Fetal head circumference (cm)	36.28 (3.3)	35.83 (1.68)	0.5^b^
- 5th minutes APGAR score	8.27 (1)	8.23 (0.7)	0.7^b^

- Data are presented as mean (standard deviation) in case of continuous variables and n (%) in case of frequencies

^a^Chi-square test

^b^Independent samples t-test

The association between fasting duration and birth outcomes was also examined, the results of which can be seen in Table 3. There was a relationship between the category of 21–29 fasting days and gestational diabetes. In addition, women from this category of fasting had less weight gain during pregnancy compared to others (*P* = 0.04).

**Table 3:** Relationship of maternal and neonatal outcome of pregnancy with number of fasting days during pregnancy

	Non (*n* = 146)	1–10 days fasting (*n* = 8)	11–20 days fasting (*n* = 14)	21–29 days fasting (*n* = 132)	*P* value
- Mode of delivery					
• Vaginal delivery	75 (51.4%)	4 (2.58%)	6 (3.87%)	28 (18.06%)	0.82^a^
• Cesarean section	71 (48.6%)	4 (2.58%)	8 (5.16%)	104 (67.09%)	
- Gestational diabetes					
• Yes	12 (8%)	0 (0%)	2 (1.29%)	2 (1.29%)	
• No	134 (91.8%)	8 (5.16%)	12 (7.74%)	130 (83.87%)	0.03^a^
- Preeclampsia					
• Yes	19 (13%)	0 (0%)	1 (0.64%)	21 (13.54%)	0.49^a^
• No	127 (87%)	8 (5.16%)	13 (8.38%)	111 (71.61%)	
- Preterm birth					
• Yes	5 (3.42%)	1 (0.64%)	0 (0%)	6 (3.87%)	0.5^a^
• No	141 (90.96%)	7 (4.51%)	14 (9.03%)	126 (81.29%)	
- Maternal weight gain during pregnancy (kg)	11.26 ± 3.06	11.25 ± 3.65	11.21 ± 2.99	10.8 ± 4.91	0.04^b^
- Low birth weight					
• Yes	4 (2.73%)	0 (0%)	0 (0%)	6 (3.87%)	0.67^a^
• No	142 (97.26%)	8 (5.16%)	14 (9.03%)	126 (81.29%)	
- Birth weight (kg)	3.35 ± 0.52	3 ± 0.0	3.33 ± 0.46	3.52 ± 1.81	0.52^b^
- Birth height (cm)	49.47 ± 2.78	47.63 ± 3.29	49.71 ± 3.56	49.42 ± 3.69	0.42^b^
- Head circumference (cm)	35.84 ± 1.67	36.25 ± 1.03	36.15 ± 1.95	36.28 ± 3.52	0.58^b^
- 5th minutes Apgar score	8.23 ± 0.77	8.13 ± 0.64	8.79 ± 0.57	8.22 ± 1.05	0.15^b^

- Data are presented as mean (standard deviation) in case of continuous variables and n (%) in case of frequencies

^a^Chi-square test

^b^One-way Anova test

Regression analysis showed that women who did not fast during the second trimester of pregnancy were 1.51 times more likely to develop gestational diabetes (OR 1.51; 95% CI 0.06, 0.74, *P* = 0.01) (Table 4).

**Table 4:** Unadjusted and adjusted relationship of pregnancy outcome with fasting behaviors

	Unadjusted		Adjusted	
	OR (95% CI)	*P* value	OR (95% CI)	*P* value
• Mode of delivery				
- Vaginal delivery	0.06 (0.83_1.05)	0.2	0.07(0.82_1.05)	0.24
- Cesarean section				
• Gestational diabetes mellitus	1.3 (0.7_0.8)	0.02	1.51 (0.06_0.74)	0.01
• Preterm labour	0.19 (0.33_4.42)	0.7	0.17 (0.32 _4.4)	0.79
• Preeclampsia	0.04 (0.51_ 2.09)	0.9	0.009 (0.49_2.04)	0.98
• Low birth weight	0.64 (0.45_8.01)	0.38	0.62 (0.44_7.86)	0.39
• Birth weight	0.1 (0.68_1.16)	0.39	0.1 (0.7_1.15)	0.41
• Birth height	0.02 (0.94_1.11)	0.49	0.03 (0.94_1.12)	0.45
• Head circumference	0.11 (0.78_1.01)	0.07	0.1 (0.79_1.02)	0.11
• 5th minutes Apgar score	0.00 (0.7_1.36)	0.99	0.01 (0.72_1.34)	0.92

All models adjusted for age, maternal education, maternal occupation, number of para, and BMI before pregnancy

As shown in Table 5, the results were found to be statistically significant when maternal fasting behaviors were cross-tabulated with questions regarding the women’s perception respecting fasting during pregnancy. Additionally, 60% of the mothers who fasted during pregnancy perceived that Ramadan fasting is compulsory for women with normal pregnancy compared to 24.3% of women in the non-fasting group. The higher number of the fasting mothers perceived that fasting is compulsory for women with high-risk pregnancy, compared with those who did not fast.

**Table 5:** Perceptions of fasting and non-fasting mothers with respect to fasting during pregnancy

	Fasted (*n* = 155)	Non (*n* = 144)	*P* value*
**-** Dose she believe that Ramadan fasting is compulsory in pregnancy for healthy woman			
• Yes	93 (60%)	35 (24.3%)	*P* = 0.000
• No	62 (40%)	109 (75.7%)	
**-** Dose she believe that Ramadan fasting is compulsory in pregnancy for high risk woman			
• Yes	28 (18.1%)	6 (4.2%)	
• No	127 (81.9%)	138 (95.8)	P = 0.000
**-** Why did not she fast in this pregnancy?			
A. She feels that she was not able to be fast due to its difficulty	- -	66 (45.8%)	
B. Fasting is not compulsory in pregnancy	–	35 (24.3%)	
C. She will compensate the fasting after pregnancy	–	32 (22.2%)	
D. Her family discourage her to be fast in pregnancy	–	6 (2.8%)	
E. She believes that fasting has negative effect on her pregnancy		4 (2.8%)	
**-** Why did she fast during pregnancy?			
A. She did not like to compensate fasting after pregnancy	96 (61.3%)	–	
B. Fasting is compulsory in pregnancy	30 (19.4%)	–	
C. She believes that fasting has not negative effect on her pregnancy	23 (14.8%)	–	
D. She was not comfort to eat in the presence of her family, so prefer to be fast	7 (4.5%)	–	
- Which discomfort did she suffer due to fating in Ramadan?			
A. Hunger or thirst	59 (38.1)	–	
B. Nausea and vomiting	47 (30.3%)	–	
C. Dizziness	59 (38.1%)	–	
D. Weakness or fatigue	2 (1.3%)	–	
Mothers who discontinued fasting (*n* = 35)			
­ Why had she discontinued fasting?		–	
A. She felt discomfort with fasting	17 (48.57%)	–	
B. Her family stopped her from fasting	8 (22.85%)	–	
C. Being advised by her obstetrician to discontinue fasting	10 (28.57%)		
**-** How did she feel regarding discontinuation of fasting?			
A. Dissatisfied	30 (85.7%)	–	
B. Satisfied	5 (14.28%)	–	

*Chi-square test

The fasting and non-fasting groups answered separate questions regarding their perception to fast or not during pregnancy. Among the women who did not fast during pregnancy, the perception of not being able to fast due to its difficulty was the most frequent reason for not fasting (45.8%). The perception that fasting is not compulsory for pregnant women was the second reason for not fasting amongst the non-fasting mothers.

The majority of the fasting women (61.3%) indicated that they fasted since they did not want to compensate for it after pregnancy. In addition, approximately 19.4% of the women who participated in Ramadan fasting had the perception that fasting during pregnancy is obligatory, 14.8% of them fasted since they believed that fasting is in not detrimental for their pregnancy, and 4.5% preferred to fast since they did not like to be watched by their family when they were eating. From the mothers who discontinued fasting during pregnancy, inconvenience with fasting was the main reason to stop fasting, and 85.7% of them were dissatisfied with failure to continue fasting for the whole Ramadan. Hunger and dizziness were the most discomfort experienced by the mothers who fasted.

## Discussion

This study was conducted to compare the pregnancy outcome and perception of women who fasted during the second trimester of current pregnancy with women who did not fast. About 80% of the women in the fasting group in this study fasted for 21–29 days during Ramadan, out of whom 38.7% completed fasting for the entire period of Ramadan. Similar fining has been reported in a study conducted by Ziaee el al. who observed that 31.7% of the mothers in their study conducted in Iran fasted for the entire Ramadan [23]. In the present study, no association was found between preeclampsia, mode of delivery, LBW, 5th minute Apgar score, newborn birth weight, height and head circumference, and fasting behaviors.

Our study is consistent with other studies which did not find an association between fasting and fetal anthropometrics measurement. In a cross-sectional study by Arab et al. in which the relationship of maternal fasting and newborn birth weight was assessed [24], it was found that Ramadan fasting did not affect the birth weight of newborns. Almond et al. found statistically insignificant decrease in the birth weight of newborns when Ramadan fasting occurred during the third trimester of pregnancy [10]. Another study conducted in Iran showed that Ramadan fasting during the third trimester of pregnancy did not alter neonatal anthropometric measurements [25].

In a prospective cohort study conducted at the UK, comprising 310 pregnant Muslim women of Asian or Asian British ethnicity, no associations were found between fasting during pregnancy and low birth weight and birth weight of the newborns [15]. In another study by Jamilian et al., no statistical difference was found between fasting and non-fasting mothers in terms of fetal heart rate, fetal movement, mode of delivery, birth weight, birth height, head circumferences, and Apgar score [26].

Examining the effect of fasting behavior on pregnancy outcomes in this study revealed that the women who did not fast during the second trimester of pregnancy were 1.51 times more likely to develop gestational diabetes. It was also found that among the women in the fasting group, those who fasted for 21–29 days during pregnancy had a lower risk of gestational diabetes compared to the other groups. This study revealed that the average weight gain during pregnancy in fasting women was lower than non-fasting group, particularly in women who fasted for more than 20 days during second trimester of pregnancy. It has been suggested that this decrease in body weight during Ramadan could be attributed to a decrease in fluid intake [27, 28]. It can also be a result of decrease in glycogen-bound water stores, extracellular volume contraction secondary to a lower sodium intake, and a moderate degree of hypohydration with little loss of body tissue [29]. There is evidence that excessive gestational weight gain in early pregnancy may increase the risk of GDM in pregnant women [30]. Although the exact mechanism of how excessive weight gain may contribute to gestational diabetes is unknown, researchers hypothesize that rapid weight gain early in pregnancy may increase insulin resistance which in turn leads to the “exhaustion” of the beta-cells in the pancreas that produce and release insulin which controls the level of glucose in the blood. This could reduce the capacity of the beta-cells to secrete adequate levels of insulin to compensate for the insulin resistance induced by the progression of pregnancy and therefore lead to the development of gestational diabetes [31]. In the studies conducted by Mirghani et al. on pregnant women who fasted intermittently during Ramadan, fasting blood glucose levels in the fasting mothers significantly decreased compared with the non-fasting mothers [13, 32]. Caloric restriction studies revealed that intermittent fasting changes the fuel selection and improves the metabolism efficiency while reduces oxidative stress [33–35]. The results of a prospective observational study by Afandi and colleagues in 2017 on 32 patients with GDM indicated that glucose levels were better in those women with GDM fasting in Ramadan than pre-Ramadan whether they were treated with diet plus metformin or diet only, and they had the highest rate of normoglycemia (90.2%); however, the higher rate of hypoglycemia was observed in the women with GDM [36].

In contrast with the present study, the results reported by Mirgani et al. showed that maternal diet restriction is associated with an increased maternal risk of gestational diabetes mellitus [37]. In a study by Al Ketbi et al., the mean random blood glucose level was significantly higher in the fasting group comparing with the control group 1 h after breaking the fast (*p* = 0.002) [38]. However, similar to other studies that assessed the biochemical changes in mothers fasting during pregnancy, association of these changes with gestational diabetes was not analyzed [3]. Moreover, most studies reporting negative impacts of prenatal Ramadan fasting were conducted without access to reported fasting data; therefore, they were unable to examine the potential role of exposure duration [10, 39, 40].

The present study gives a clear insight into the perceptions of fasting among Iraqi women during pregnancy. It has been revealed that the decision to fast during pregnancy was negatively associated with the mother’s educational level and occupation, such that with an increase in the education levels and frequency of working, fewer mothers fasted. This finding is consisted with the results found by Nusrat and colleagues in Iran who observed that a higher number of the mothers in the fasting group had lower education levels [41].

A higher number of mothers in the fasting group perceived that fasting during pregnancy was compulsory for healthy and non-healthy mothers, comparing with mothers who did not fast. This indicates that they were unaware of the permissibility and exemption of fasting in Islam during pregnancy, or there is a lack of communication by the nurses and other healthcare staff to provide efficient and relevant prenatal information for pregnant women [3].

This study suggested that participation to Ramadan fasting was mostly associated with the fact that the mother were reluctant to repay the fasting after pregnancy. The same response was observed by Pakistani women when they were asked about their reason for fasting during pregnancy [5]. It may indicate that women would like to fast with their families rather than doing this alone later. Uncomfortable feeling while they were eating in the presence of family was found as another reason for fasting in this study. On the other hand, just 2.8% of the mothers in the non-fasting group were encouraged not to fast during pregnancy. It may associate with the role of gender in Iraq where the role of women is diminished in the family and society according the prevailing culture in society [42]. Research has revealed that husband’s opinion affects a woman’s decision to fast, so that women are less likely to fast if their husband supports and encourages them not to fast [43].

In Islam, pregnant women are allowed not to observe fasting if they are concerned about risking their and their fetus’s health [5, 44]; however, just 2.8% of the non-fasting mothers did not fast because of this belief. On the other hand, just 28.57% of the fasting mothers stopped fasting when they were advised by the obstetrician. This could reflect how they perceive the rule for fasting in pregnancy or how their healthcare staff failed to provide them with relevant and efficient information.

Hunger or thirst was the most discomfort experienced by the fasting mothers during pregnancy. Metzger and his co-workers first defined accelerated starvation signs in pregnant women when skipping breakfast after nocturnal fasting in comparison with non-pregnant women [45].

To the best of the authors’ knowledge, the present study is the first investigation that has been conducted in Iraq in order to assess the perspectives and pregnancy outcomes of maternal Ramadan fasting; however, this study should be considered in light of its limitations. Since interview was performed at the time of birth, the possibility of recall bias for the data regarding fasting increased. A further limitation may be the external generalizability of these results to wider Muslim populations which may comprise different traditions, dietary habit, and beliefs about fasting. Another limitation of the present study is it only included women who fasted during the second trimester of pregnancy, and the effect of fasting on pregnancy in different trimester of pregnancy cannot be compared.

## Conclusion

This study revealed that some of the pregnant Muslim women did not fully recognize their right of being exempted from fasting by the Islamic law. It is critical to raise the mothers’ knowledge regarding this issue by establishing official guidelines. Health care providers also have an important role in promoting the mothers’ knowledge regarding fasting during pregnancy. This study showed that fasting during the second trimester of pregnancy decreased the risk of gestational diabetes. Since there are conflicting findings regarding the effect of fasting on pregnancy outcomes, further studies with larger sample sizes are required to replicate the findings of the present study, and effects of fasting on pregnancy outcomes should also be investigated during the first and second trimesters of the pregnancy.

## Additional file

Additional file 1: Questionnaire assessing perspectives and pregnancy outcomes of maternal Ramadan fasting in the second trimester of pregnancy. (DOCX 38 kb)

## Abbreviations

BMI: Body mass index
CI: Confidence interval
GDM: Gestational diabetes
LBW: Low birth weight
One-way ANOVA: One-way analysis of variance
OR: Odds ratio
SD: Standard deviation
