# Correction to: Perspectives and pregnancy outcomes of maternal Ramadan fasting in the second trimester of pregnancy

**DOI:** 10.1186/s12884-019-2647-2

**Published:** 2019-12-09

**Authors:** Kolsoom Safari, Tiran Jamil Piro, Hamdia Mirkhan Ahmad

**Affiliations:** 1 Department of Midwifery, College of Nursing, Hawler Medical University, Erbil, Iraq; 2 Department of Basic Science, College of Basic Science, Hawler Medical University, Erbil, Iraq

**Correction to: BMC Pregnancy Childbirth (2019) 19:128**

**https://doi.org/10.1186/s12884-019-2275-x**

Following publication of the original article [1], the author notified us about the incorrect values on Table 4.

**Table 4:** Unadjusted and adjusted relationship of pregnancy outcome with fasting behaviors

	Unadjusted		Adjusted	
	OR (95% CI)	*P* value	OR (95% CI)	*P* value
• Type of birth				
- Vaginal delivery	0.93 (0.83_1.05)	0.2	0.92 (0.82_1.05)	0.24
- Cesarean section				
• Gestational diabetes mellitus	0.26 (0.07_0.8)	0.02	0.2 (0.06_0.74)	0.01
• Preterm labour	0.68 (0.33_4.42)	0.7	0.8 (0.32 _4.4)	0.79
• Preeclampsia	0.9 (0.51_ 2.09)	0.9	0.91 (0.49_2.04)	0.98
• Low birth weight	1.1 (0.45_8.01)	0.38	1.1 (0.44_7.86)	0.39
• Birth weight	0.6 (0.68_1.16)	0.39	0.1 (0.7_1.15)	0.41
• Birth height	1.04 (0.94_1.11)	0.49	1 (0.94_1.12)	0.45
• Head circumference	0.89 (0.78_1.01)	0.07	0.9 (0.79_1.02)	0.11
• 5th minutes Apgar score	1.03 (0.7_1.36)	0.9	0.9 (0.72_1.34)	0.92

All models adjusted for age, maternal education, maternal occupation, number of para, and BMI before pregnancy

The true **Odds** for gestational diabetes (GDM) is 0.2 not 1.51. Therefore, in addition to correcting the values for OR in the Table 4, the following sentence in the published article:

Women who did not fast during the second trimester of pregnancy were 1.51 times more likely to develop gestational diabetes.

Should be replaced by:

Women who fasted during the second trimester of pregnancy were 0.8 times less likely to develop gestational diabetes.

Correcting the **OR** value will not affect other part of the study in any way. It also prevents misinterpretation of our findings, and clearly shows the main result of this study “negative relationship between fasting during pregnancy and risk of GDM”, which is approved by other tests used in this study.

The authors apologize for any inconvenience caused by this error.
