# Supplementary material for: Perspectives and pregnancy outcomes of maternal Ramadan fasting in the second trimester of pregnancy
Source: BMC Pregnancy Childbirth. 2019 Apr 15;19:128. doi: 10.1186/s12884-019-2275-x (PMC6466666; doi:10.1186/s12884-019-2275-x)
Supplement: Supplementary file 1 — Questionnaire assessing perspectives and pregnancy outcomes of maternal Ramadan fasting in the second trimester of pregnancy. (DOCX 38 kb) [file 12884_2019_2275_MOESM1_ESM.docx]

**Questionnaire**

**Ethical number: (Ref 32N/2017)**

**Title of the study:** Perspectives and pregnancy outcomes of maternal Ramadan fasting in the second trimester of pregnancy

- **I confirm that I have read and understood the information about the project as provided in the Participant Information Sheet dated (------------------).**
- **I confirm that that I have had the opportunity to ask questions and the researcher has answered any questions about the study to my satisfaction.**
- **I understand that my participation is voluntary and that I am free to withdraw from the project at any time, without having to give a reason and without any consequences.**
- **I understand that I can withdraw my data from the study at any time.**
- **I understand that any information recorded in the investigation will remain confidential and no information that identifies me will be made publicly available.**
- **I consent to use of the data in research, publications, sharing and archiving as explained in the Participant Information Sheet**
- **I consent to being audio/video/ interviews being recorded as part of the project (delete if not being used] Yes/No**
- **I agree / do not agree (delete as appropriate) to take part in the above study.**

**Name of Participant --------------------------Date-----------------------------Signature**

**Researcher-----------------------------Date------------------------------------ Signature**

| **Part 1: Socio-demographic Data** |
| --- |
| 1. **Age:** |
| 1. **Marriage Age:** |
| 1. **Education level (years) :** |
| 1. **Residency: □Citizenship of Erbil □ Immigrant** |
| 1. **Monthly income: …….. $** |
| 1. **Occupation: □Housewife □Employee** |
| 1. **Weight before pregnancy:**   **Weight at the time of delivery:**  **Height :**  **BMI:**  **Weight gain during pregnancy:** |
| **Part 2: Obstetrical and past medical History** |
| 1. **Number of gravida:** 2. **Number of para:** 3. **LMP:** 4. **Gestational Age:** |
| **Part 3: Questions regarding fasting** |
| 1. **Previous history of fasting during pregnancy: □ yes □ no** 2. **Does she believe Ramadan fasting is compulsory in pregnancy for healthy women?□yes □ no** 3. **Does she believe Ramadan fasting is compulsory in pregnancy for high risk women?□yes □no**   **5. Does she satisfy with fasting during pregnancy?□ yes □ no**   1. **Where does she collect information regarding Ramadan fasting in pregnancy?**   **□Mullah □Relatives □physician □Nurse □Media**  **7. Has she ever consulted regarding fasting during pregnancy by her physician?**  **□ yes □ no** |
| 1. **Did she fast during second trimester of the current pregnancy?**   **□**Yes **□**No |
| **If no,**   1. **Why did not she fast in this pregnancy?** 2. She feels that she was not able to be fast due to its difficulty 3. Fasting is not compulsory in pregnancy 4. She will compensate the fasting after pregnancy 5. Her family discourage her to be fast in pregnancy 6. She believes that fasting has negative effect on her pregnancy |
| **If yes,**   1. **Why did she fast during pregnancy?** 2. She did not like to compensate fasting after pregnancy 3. Fasting is compulsory in pregnancy 4. She believes that fasting has not negative effect on her pregnancy 5. She was not comfort to eat in the presence of her family, so prefer to be fast 6. **How many days did she fast during second trimester of this pregnancy?** |
| **If she discontinued fasting,**   1. **Why had she discontinued fasting?** 2. She felt discomfort with fasting 3. Her family stopped her from fasting 4. Being advised by her obstetrician to discontinue fasting |
| 1. **Which discomfort did she suffer due to fating in Ramadan?** 2. Hunger or thirst 3. Nausea and vomiting 4. Dizziness 5. Weakness or fatigue |
| 1. **Was she advised by her physician/ nurse to be fast in this pregnancy**? **□ yes □ no** 2. **Who did encourage her most to be fast in this pregnancy?** |
| **Part 4: Birth Outcome** |
| 1. **Maternal outcomes of the pregnancy**: **□**Gestational diabetes **□** Preeclampsia   **□** Preterm labour |
| 1. **Anthropometric measurement of the newborn** 2. **Weight:** 3. **Height:** 4. **Head circumference:** 5. **5^th^ minutes Apgar score:** |
